# Precision-Oriented Sensing and Multimodal Visuo-Haptic Interaction for Fine Manipulation in Biomedical Engineering and Assistive Applications

**DOI:** 10.3390/s26216792

**Published:** 2026-10-27

**Authors:** Susanna Cifani, Marco Raoul Marini, Paolo Bottoni, Atsushi Nakamura, Kamen Kanev, Luigi Cinque

**Affiliations:** 1 Department of Computer Science, Sapienza University of Rome, 00161 Rome, Italy; cifani@di.uniroma1.it (S.C.); marini@di.uniroma1.it (M.R.M.); bottoni@di.uniroma1.it (P.B.); cinque@di.uniroma1.it (L.C.); 2 Research Institute of Electronics, Shizuoka University, Hamamatsu 432-8011, Shizuoka, Japan; nakamura.atsushi@shizuoka.ac.jp; 3 Faculty of Business and Information Technology, Ontario Tech University, Oshawa, ON L1G 0C5, Canada

**Keywords:** virtual reality, haptics, force feedback, data glove, carbon nanotube sensors, hybrid interfaces

## Abstract

Virtual Reality (VR) simulations in high-stake domains, such as surgical training or robotic teleoperation, require both high visual fidelity and accurate force feedback to facilitate embodied cognition and prevent errors. However, current commercial haptic devices impose a critical trade-off: while they provide force feedback, their embedded sensors are known to be prone to latency, hysteresis, and tracking noise, which may limit the precise finger flexion tracking necessary for fine manual dexterity. Conversely, data gloves designed specifically for precise finger tracking offer finer input granularity but lack active force feedback capabilities. To address these limitations, we introduce a hybrid architecture that simultaneously deploys precision-oriented data gloves and haptic gloves, presenting a proof-of-concept user study to examine the performance benefits and trade-offs of combining precision-oriented tracking with direct haptic feedback during the fine manipulation of delicate virtual objects. From our findings, we derive insights into optimizing multimodal visuo-haptic interaction for future biomedical and assistive environments.

## 1. Introduction

Virtual Reality (VR) has gained a relevant role in skill acquisition in high-stakes domains. Fields such as surgical training [1,2,3], robotic teleoperation [4], and industrial assembly [5,6] increasingly rely on immersive simulations to develop practical skills in risk-free environments. In these domains, visual fidelity alone appeared to be insufficient [7]: to acquire complex motor skills, users must be able to “feel” the virtual environment [8]. This necessity is grounded in the theory of embodied cognition, which posits that learning is deeply rooted in the sensorimotor system. Since haptic feedback facilitates “cognitive offloading” [9,10], accurate force feedback is a functional requirement to manipulate delicate instruments without causing damage [11,12].

Despite these theoretical advantages, current commercial haptic devices impose a severe technical trade-off. While they provide force feedback, their embedded sensing mechanisms (such as cable extensions or resistive bend sensors) are known to introduce significant tracking noise, hysteresis and latency, which may limit the precise finger flexion tracking required for fine manual dexterity [13]. Conversely, data gloves designed for precise finger tracking offer finer input granularity but lack active force feedback capabilities.

Building upon our established research trajectory in psychomotor training [2] and assistive communication [14,15], our previous work addressed motion tracking and neuromuscular stimulation through advanced data glove technologies [16]. While those foundational systems successfully integrated precision-oriented tracking (such as Yamaha Data Gloves), a critical next step lies in augmenting their functionality through the introduction of direct haptic feedback. To this end, the present study explores the simultaneous deployment of precision-oriented data gloves and haptic gloves within a hybrid architecture. Since direct joint-level sensing is not subject to the cable friction and hysteresis discussed above, we expect it to provide more precise finger tracking, and we examine how this expected advantage translates into fine manipulation at the user level.

## 2. Background and Related Work

We review work on various aspects touched upon in the paper, from the theoretical grounding of haptic feedback in embodied learning (Section 2.1), to the technical limitations of current commercial haptic hardware (Section 2.2) and existing approaches decoupling finger tracking from force feedback (Section 2.3). We situate our contribution within our own research trajectory, covering assistive and psychomotor training applications (Section 2.4), specifically, neuromuscular stimulation and rehabilitation systems (Section 2.5).

### 2.1. Haptics and Embodied Learning

The integration of haptic feedback in VR is grounded in the theory of embodied cognition, which posits that cognitive processes are deeply rooted in the sensorimotor system [9]. Research indicates that haptic feedback facilitates cognitive offloading, allowing learners to process information through their bodies rather than visual working memory alone [10]. This is relevant to procedural knowledge, where resistance and texture sensations help build muscle memory. However, while VR training offers a safe environment for learning and building confidence, replicating the task efficiency of real-world practice remains a technological challenge. For example, a recent comparison of VR-based training against physical execution [17] observed that task completion times in the virtual environment were nearly twice those of the physical counterpart. This specific performance gap was attributed to a combination of user unfamiliarity and intrinsic interface limitations, with the study noting that extensive fine-tuning of the haptic gloves was required to mitigate mechanical discrepancies. Such findings highlight a critical issue framed within evolutionary cognitive theory [18]. Fundamental actions like grasping are classified as “biologically primary knowledge”, but when hardware fidelity is insufficient, the interface fails to be transparent, transforming these intuitive actions into cognitively demanding tasks.

### 2.2. Commercial Haptic Hardware Trends

Current commercial hardware struggles to match the dexterity of the human hand, particularly in terms of finger flexion tracking. While the market is currently led by solutions such as SenseGlove Nova [19,20], Manus Prime [21], and HaptX [22], the embedded sensing technologies in these devices often fail to provide the precision required for fine dexterity. Specifically, devices relying on cable extension measurements (such as SenseGlove) are susceptible to mechanical friction and hysteresis, causing discrepancies in bending detection. Similarly, gloves utilizing resistive bend sensors (such as Manus) are prone to signal drift over time and non-linear outputs that require frequent recalibration. Comprehensive reviews of haptic technologies confirm that most gloves rely on these indirect sensing methods, which introduce drift and lack accuracy [13]. This leads to what has been described as the “uncanny valley of haptics” [23]: when high-quality visual feedback is paired with low fidelity or asynchronous tactile feedback, the user’s sense of agency breaks.

### 2.3. Decoupling Finger Tracking from Force Feedback

A natural response to these sensing limitations is to separate finger tracking from force-feedback actuation, so that each can rely on the most suitable technology. Early systems followed this principle by mounting a cable-driven exoskeleton over a bend-sensor data glove, as in the CyberGlove/CyberGrasp combination [24]: this provided per-finger kinesthetic feedback on top of dedicated sensing, but required bulky, tethered hardware that limited wearability. More recent research prototypes integrate distinct sensing and actuation subsystems within a single custom device, such as soft robotic gloves pairing IMU-based finger tracking with motor-tendon force feedback for VR rehabilitation [25], or low-cost force-feedback gloves for teleoperation [4]. Similar integrated designs combine stretchable sensors with shape memory alloy actuation [26] or with pneumatic restrictive actuation and per-user finger calibration [27]. These systems allow tracking accuracy to be validated independently, typically through bench tests or comparison with optical motion capture [28,29], but rely on ad hoc hardware that is not readily available. A third strategy avoids instrumenting the fingers altogether, pairing optical hand tracking with wearable haptic devices [30]; however, camera-based tracking depends on line of sight and can fail to recognize the hand when it is covered by a haptic glove [31]. Overall, existing approaches have to balance sensing fidelity with wearability and hardware availability, while decoupling with off-the-shelf components remains largely unexplored.

### 2.4. Assistive and Psychomotor Training Applications

Given these trade-offs, high-stakes domains often prioritize input fidelity over force feedback. Hence, specialized data gloves employing graphene-based piezoresistive sensors [32] or carbon nanotube (CNT) strain sensors [33] are frequently adopted in critical scenarios such as medical training and assistive communication. Our prior work has extensively explored soft-sensor wearables, haptic feedback, and multi-device interaction frameworks to address motor rehabilitation, psychomotor training and assistive communication. Our initial efforts focused on mitigating non-compliance in physical therapy by combining haptic wearables with gamification and social robotics [34]. Using a Yamaha Data Glove embedded with CNT strain sensors to capture 12 Degrees of Freedom (DoF) across the wrist and phalanges, we mapped biomechanical telemetry to a virtual crane exergame paired with an ASUS Zenbo Junior robot for real-time instructional feedback [34]. To extend soft-sensor tracking into fine manual dexterity, lightweight CNT strain sensors embedded in fabric gloves were integrated into the Virtual Anesthesia Crisis Scenario Builder (ACSB) platform [2]. Replacing traditional handheld VR controllers, this glove-based tracking enabled trainees to practice delicate clinical procedures—such as manipulating medication vials and maintaining exact syringe needle angles to prevent coring—establishing a framework for psychomotor-driven medical simulations [2]. To address severe sensory impairments, soft-sensor gloves were adapted to capture subtle metacarpophalangeal (MCP) and interphalangeal (IP) joint movements without bulky hardware, enabling contact-free single-handed Mobile Malossi signing transmitted wirelessly via XBee for remote messaging and direct control of mobile assistive robots [15]. To complement gesture recognition with direct haptic perception, we prototyped a two-way communication framework featuring a snap-button entry input glove paired with a receiving glove equipped with 12 mm circular piezo actuators (280 Hz, 15 V AC) delivering localized tactile signals across mirrored anatomical positions [14]. From these multi-device deployments, we also formalized specialized user experience (UX) guidelines to mitigate sensor noise, manage physical cabling constraints, blend physical–virtual environments, and leverage auditory rhythms for human-robot movement synchronization [35].

### 2.5. Neuromuscular Stimulation and Rehabilitation Systems

Within this research trajectory, motor rehabilitation motivated a more targeted line of work, centered on active neuromuscular stimulation. We expanded soft-sensor tracking into active neuromuscular stimulation by integrating an 11-sensor CNT data glove on a reference hand with a 24-channel Functional Electrical Stimulation (FES) system driven by multi-pad wet gel electrodes (0–50 mA) [16]. This bilateral framework maps joint flexion vectors to pre-calibrated look-up tables via normalized Euclidean distance calculations, automatically applying biphasic constant-current pulses to evoke mirror-symmetric finger bending in a target hand while validating movement via a secondary glove for post-stroke upper-limb rehabilitation and FES-assisted signing [16]. Building upon this foundation, our current work moves beyond feasibility to an exploratory evaluation, aiming to explore, at the user level, the benefits and costs of precision-oriented tracking in the fine manipulation of delicate virtual objects within biomedical and assistive contexts.

**Our contribution.** At this stage, rather than attempting a full, complex hardware integration with an active clinical rehabilitation system—which remains a promising direction for future research—we propose a foundational proof of concept. By investigating this hybrid configuration, we aim to evaluate its potential for augmenting the functionality and precision of rehabilitation and assistive systems. Through an exploratory evaluation of fine manipulation of delicate virtual objects, this work explores, at the user level, the performance benefits and trade-offs of precision-oriented tracking combined with haptic feedback, providing valuable insights for future assistive environments. Unlike previous decoupling approaches (see Section 2.3), the proposed architecture combines two off-the-shelf gloves, integrating them in software through a kinematic override, and compares the result directly with the haptic glove’s native sensing. A preliminary version of this architecture was presented in [36].

## 3. Proposed Method

This study compares two distinct hardware configurations: the standard setup of a commercial haptic glove against an augmented configuration of the same system, within the same virtual reality environment. Both setups utilize a **Meta Quest 3** as the Head-Mounted Display (HMD). While the current implementation focuses on the user’s right hand, the architecture is designed to be fully scalable to bimanual scenarios. The following subsections detail the baseline commercial setup and the proposed hybrid augmentation.

### 3.1. Standard System (Baseline)

**Figure 1:**
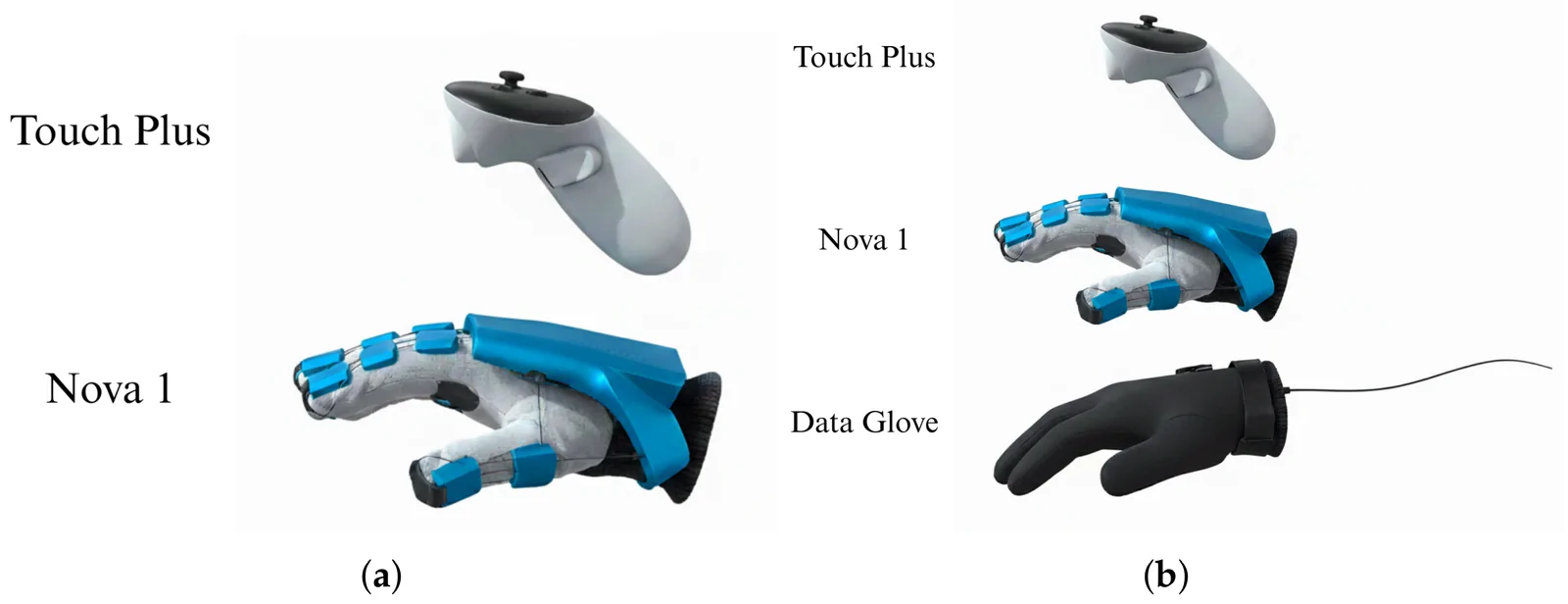
Overview of the two experimental hand hardware configurations. (**a**) The Standard system. (**b**) The Hybrid system.

The standard configuration corresponds to the typical usage of haptic gloves and serves as the baseline for the study. The user wears a **SenseGlove Nova 1**, equipped with a **Meta Touch Plus controller** mounted on the dorsal side (see Figure 1a). The controller acts purely as an IMU (combined with the headset’s optical tracking system) to provide 6-DoF spatial positioning of the hand (wrist transform), and its native haptic vibration capabilities are disabled. The Nova 1 communicates with the headset via Bluetooth.

The glove tracks finger flexion by measuring the linear displacement of internal cables routed along the dorsal side of the thumb, index, middle and ring fingers, while pinky flexion is not measured but coupled to that of the ring finger. The physical contact between the moving cables and their guiding sheaths induces Coulomb friction: this can lead to mechanical hysteresis [8], where the measured cable displacement lags behind actual joint movements during rapid flexion–extension cycles [11]. The glove provides force feedback by applying mechanical brakes to these cables. Upon virtual collision, an electrical current energizes an internal electromagnetic actuator that instantly clamps the cable mechanism via friction, exerting a passive braking force of up to 20 N per finger (excluding the pinky), physically restricting further cable extension [37]. The braking force is driven by a normalized force-feedback command (in the interval [0, 1]) sent by the SDK, and scales proportionally with it. Hence, the cable-based measurement may be prone to inherent friction and signal noise, limiting the overall accuracy of finger tracking [38].

### 3.2. Hybrid System

The second configuration adopts the hybrid hardware architecture which physically layers a precision-oriented data glove underneath a commercial haptic glove, thus decoupling the tracking of input from force feedback output. As shown in Figure 1b, the user wears a **Yamaha Data Glove** (11 CNT strain sensors covering the MCP and PIP joints of all five fingers (IP for the thumb), plus an additional thumb sensor) underneath the SenseGlove Nova 1. Despite the double-gloving (a thin plastic liner was worn between the two layers to reduce friction), the inner data glove is made of a thin, stretchable fabric that minimally impacts overall comfort and preserves the user’s baseline dexterity. The key divergence from the baseline lies in the input source: the inner glove employs carbon nanotube (CNT) thin-film strain sensors to measure flexion directly at the finger joints [33], bypassing the structural limitations of external cables.

**Figure 2:**
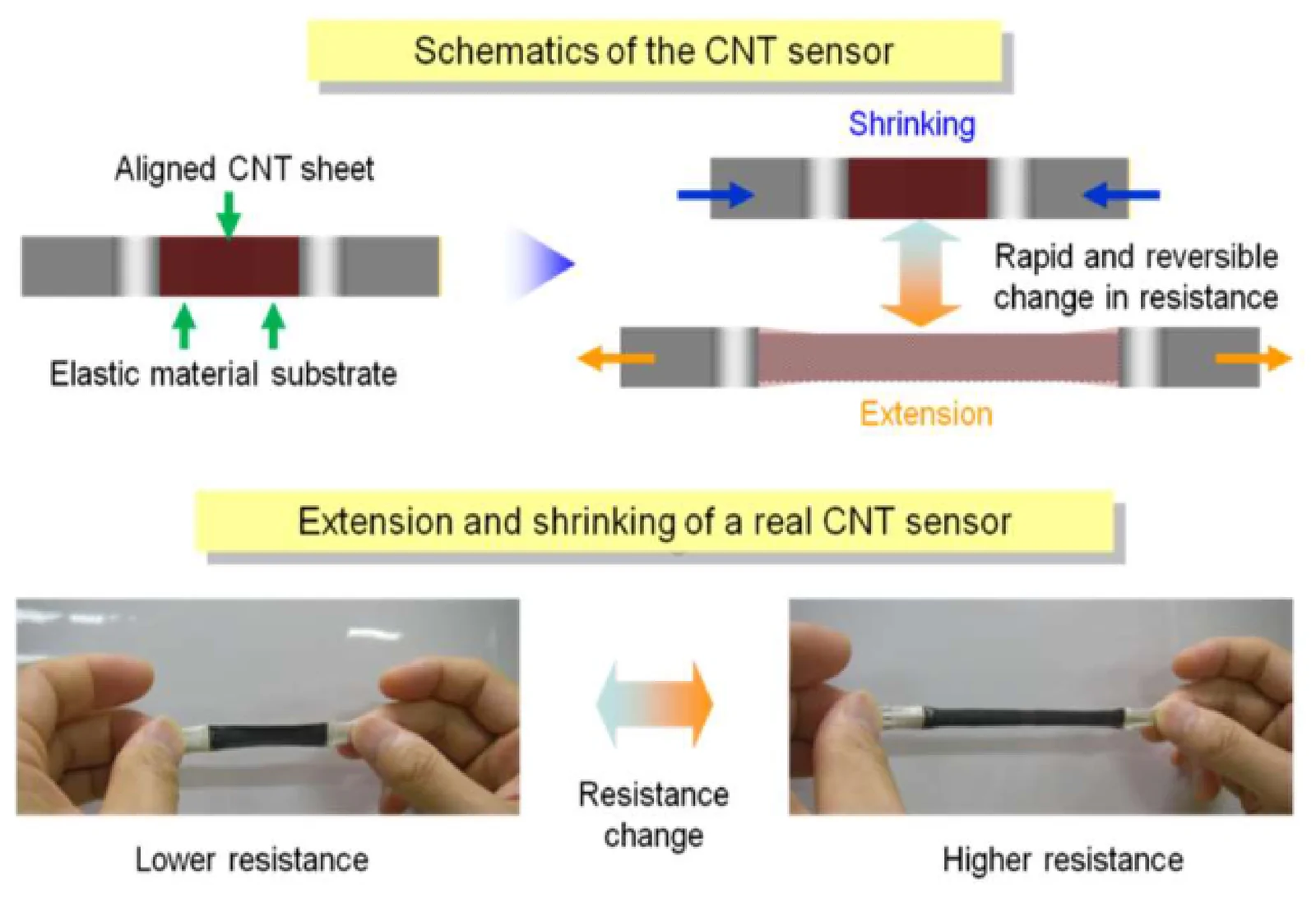
Expansion-contraction properties of the stretchable CNT sensors. Reproduced under the terms and conditions of the Creative Commons Attribution license [33].

When a CNT-based composite is mechanically deformed through finger bending, the underlying nanotube network undergoes structural changes (see Figure 2). This leads to measurable variations in its electrical resistance:

- **Stretching**: Increases the distance between CNTs, leading to an increase in resistance.
- **Compression**: Decreases the distance between CNTs, hence decreasing resistance.

This yields a measurable resistance change, described by the relation:

$$\frac{\Delta R}{R_{0}}=f\left(\varepsilon \right)$$

where ΔR is the resistance variation, $R_{0}$ is the baseline initial resistance, and ε is the mechanical strain.

The sensitivity of this piezoresistive behavior is quantified by the Gauge Factor (GF):

$$GF=\frac{\Delta R/R_{0}}{\Delta L/L_{0}}$$

where $R_{0}$ is the initial resistance, ΔR is the variation in the resistance, $L_{0}$ is the initial length and ΔL is the variation between the initial length and the elongated length.

While conventional metal strain gauges exhibit a Gauge Factor typically around 2, the CNT sensors embedded in the data glove developed by Yamaha possess a GF of **approximately 10.5**, calculated via linear approximation [33] and a sensing delay below 15 ms. This high sensitivity stems from a combination of changes in contact resistance and electron tunneling within the CNT network under deformation.

Unlike the external exoskeleton design of Nova 1, where tracking cables slide within rigid guiding sheaths and adapt mechanically via adjustable straps, the CNT sensors are integrated within the stretchable fabric. Consequently, the raw resistance signals are highly susceptible to individual hand anatomy and anthropometric variations. For instance, in smaller hands (e.g., participants with shorter fingers), where the fingertips do not fully reach the glove’s endpoints, fabric stretching does not scale linearly with joint movement.

To resolve this constraint, we developed and implemented a dedicated software calibration within the input pipeline. During the initialization phase, a customized script samples the raw resistance values (*R*) at two user-specific boundary conditions: maximum extension (flat hand) and maximum flexion (fist). The boundary parameters are then used to perform real-time linear interpolation between the two calibration extremes, mapping the normalized resistance scaling directly onto the virtual hand’s joint angles. This two-point linear mapping is a simplification of the fabric’s intrinsically non-linear stretch behavior: errors are therefore expected to be the largest at intermediate flexion angles, and sensor hysteresis and drift over the session were not quantified in this study (Section 7.4).

**Figure 3:**
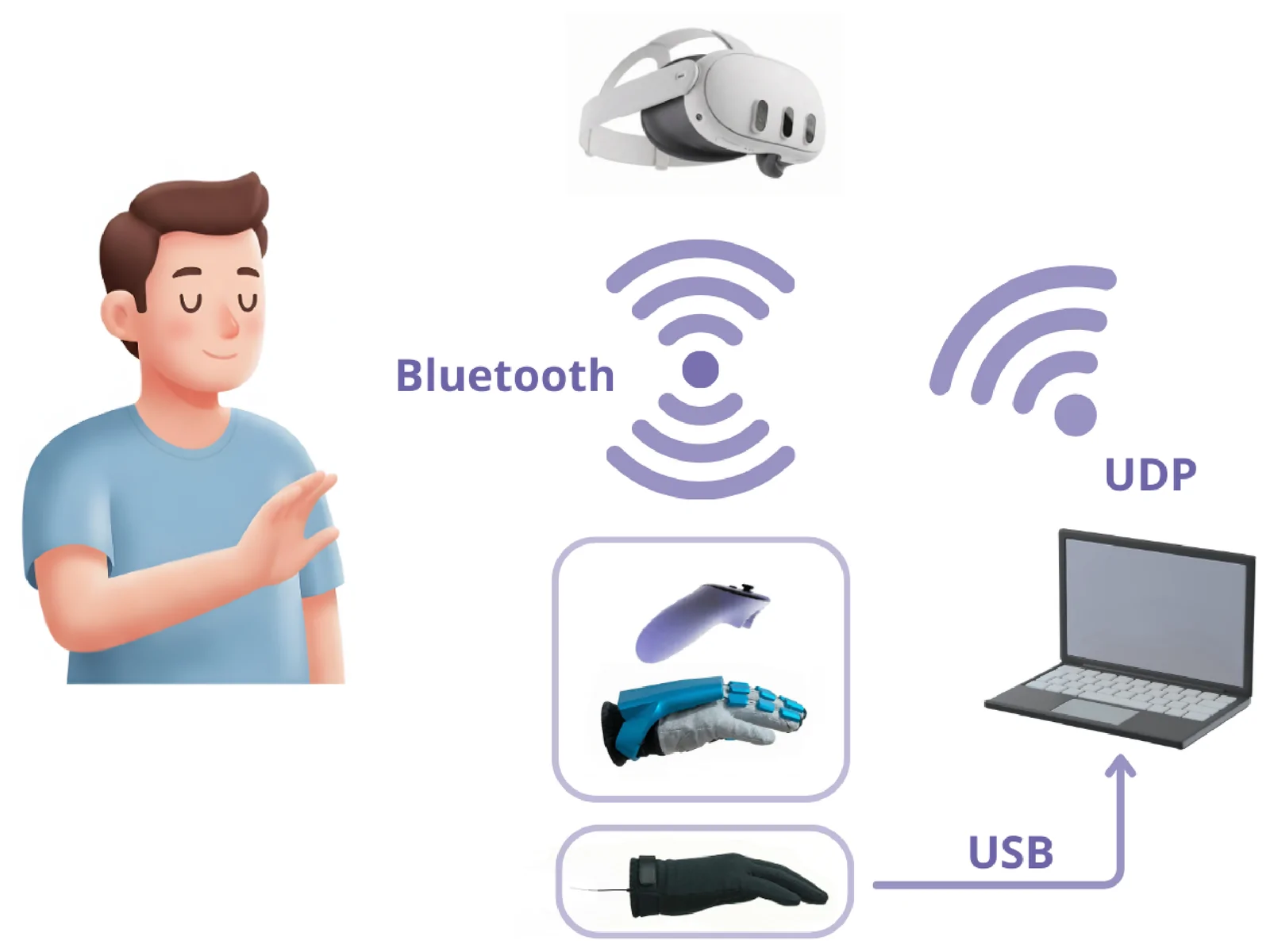
Hardware architecture and real-time data flow pipeline of the Hybrid system, showing the USB connection from the data glove to the workstation and subsequent UDP streaming to VR headset. The Nova 1 communicates with the headset via Bluetooth.

To operationalize this setup, we adopted the system architecture shown in Figure 3.

The hardware consists of the data glove, the haptic glove, a VR workstation (PC), a Meta Quest 3 headset and its Touch Plus controller. Finger flexion data from the data glove is captured via USB on the PC and subsequently streamed via UDP to the Unity 6 engine (v6000.0.27f1) application running on the headset. This pipeline allows us to inject the data glove’s flexion data directly into the simulation code (adapted from the SenseGlove SDK [39]), effectively bypassing the haptic glove’s native input. Although the hybrid architecture introduces an intermediate relay step (PC processing and local UDP streaming) compared to the direct Bluetooth pairing of the standard configuration, the delay due to data transmission is negligible. The streaming pipeline operated over an isolated, dedicated 5 GHz Wi-Fi local network deployed in the test area, exclusively serving communication between the workstation and the headset.

Under these conditions, the UDP network transmission latency was measured using a bidirectional round-trip time (RTT) timestamping procedure: network timestamps were logged for runs of 10,000 consecutive UDP packets exchanged between the host PC and the Meta Quest 3 over the dedicated connection, repeated across multiple test sessions. This yielded an average one-way transmission latency consistently below 3 ms with negligible packet loss. The overall end-to-end input latency was analytically estimated rather than directly measured: factoring in the measured network transmission (<3 ms), the hardware sensing delay of the CNT sensors (below 15 ms [33]) and the 90 Hz simulation loop (11.1 ms fixed timestep), the cumulative nominal input latency is expected to remain on the order of $10^{-2}$ s (∼25–30 ms), a timescale nominally consistent with perceptual thresholds for visuo-haptic temporal integration.

The software pipeline implements a kinematic override: the virtual hand is driven exclusively by the flexion data from the data glove, while the Nova 1’s native tracking is ignored. Both configurations share the same wrist pose (position and orientation of the hand in virtual space), provided by the Touch Plus controller through the SenseGlove SDK with its default controller-to-wrist offset, without per-user spatial co-registration, and the same virtual hand model: only the finger joint angles are replaced. Since the virtual hand model was not scaled to individual users and was identical in both configurations, any residual mismatch between physical and virtual finger geometry affected both setups equally. In this setup, the Nova 1 acts purely as a passive actuator: the physics engine calculates collisions based on the data provided by the Yamaha Data Glove and, when a virtual interaction is detected, the system triggers the mechanical brakes on the haptic glove to provide force feedback. Such decoupling is relevant for real-time training simulations, where accurate hand tracking and precise collision detection pipelines are strictly required to filter out accidental interactions during intensive manual routines [40].

## 4. Experimental Setup

We describe the environment in which the user tests took place, and provide details on the participants, the tasks involved, and the observed metrics.

### 4.1. Virtual Environment and Tasks

To evaluate user performance with the two systems, we designed a custom gamified interaction scenario using Unity 6 (v6000.0.27f1), involving three virtual objects. The three virtual objects were assigned distinct simulated behaviors (rigid, brittle, and compliant), so that each task stresses a different aspect of hand–object interaction [41]. Each object was programmed with distinct physical behaviors to evaluate three specific user skills (**transport of a rigid fragile object**, **grip closure control on a brittle object**, and **ability to maintain a stable grasp on a compliant object**) across both hardware configurations.

The interaction logic is built upon the physics-based grasping logic of the SenseGlove SDK (v2.9.0), where the virtual fingers are driven by the specific input data described in Section 3.1 and Section 3.2. A grasp is activated when the thumb and at least one other finger are in contact with the object, with a penetration of at least 2 mm to prevent spurious engagements. Physics was simulated at 90 Hz (fixed timestep 0.0111 s), matching the display refresh rate of the Meta Quest 3. Force-related parameters are expressed in the SDK’s normalized units, and all object parameters were tuned empirically in pilot tests to produce plausible behavior, rather than calibrated against real-world physical values. Participants performed three sequential manipulation tasks utilizing these objects: (1) Fragile Object Transport with the glass vial; (2) Grip Closure Control to crack the egg inside a target zone without breaking it beforehand; and (3) Grip Adaptation to hold the soft snake.

### 4.2. Fragile Object Transport (The Glass Vial)

**Figure 4:**
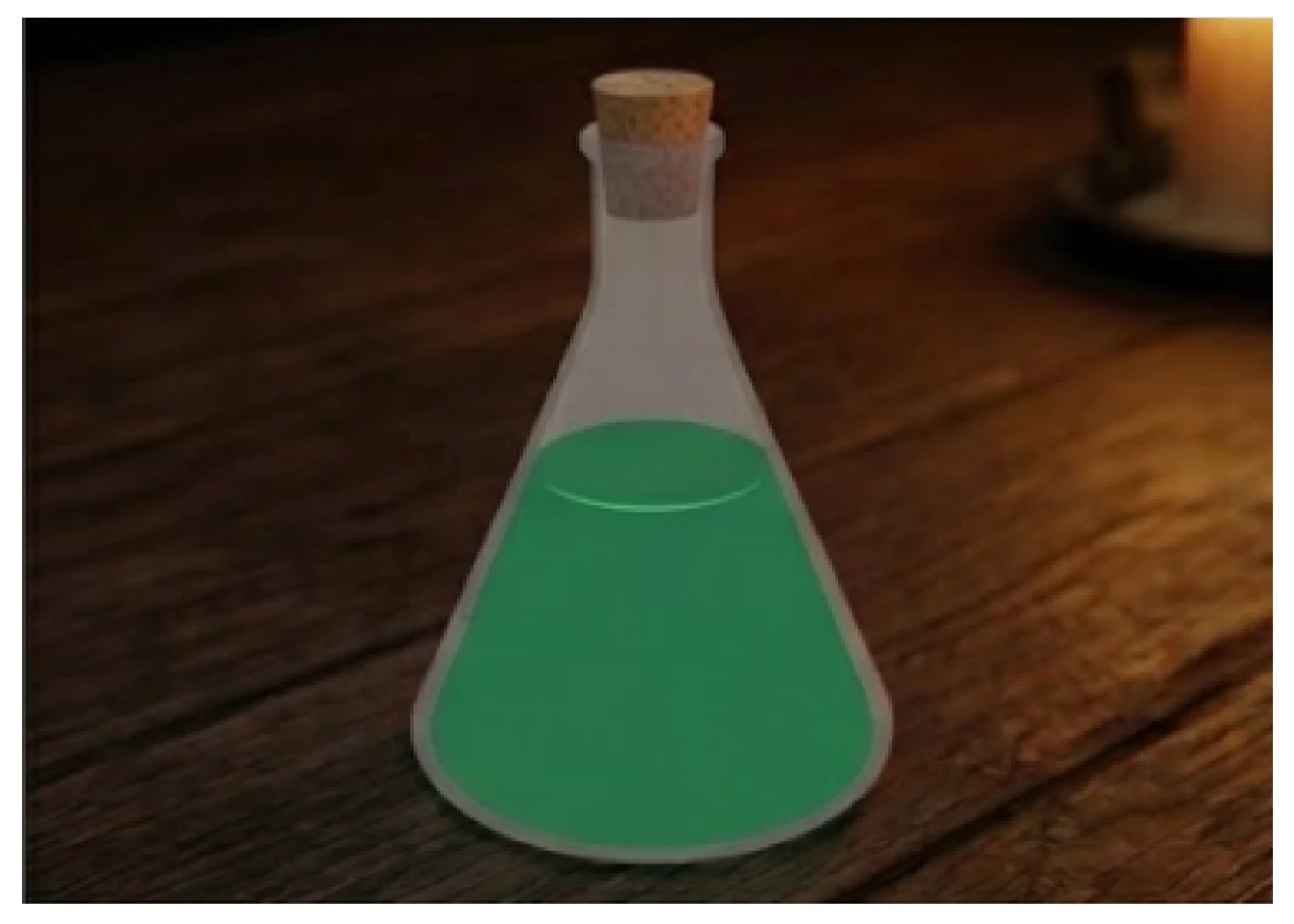
Glass Vial.

To evaluate the user’s ability to grasp and transport a rigid fragile object, we tested the manipulation of a virtual glass vial (see Figure 4).

The failure condition here is governed by the *collision velocity* against environmental colliders (e.g., table, floor). A threshold of 1.2 m/s on the impact velocity was defined to simulate the fragility of glass: if the user manipulates the vial with abrupt movements or drops it, causing an impact velocity exceeding the threshold, the object shatters. This scenario probes whether users can maintain reliable control of a fragile object with each configuration. Signal noise can cause unintended finger extension (dropping the vial) or jitter (registered as false high-velocity impacts), thereby testing whether the user can trust the virtual hand for delicate maneuvers. This task was conceptually motivated by clinical procedures requiring precise object handling under time pressure, such as the manipulation of medication vials in our prior anesthesia crisis simulation work [2], where excessive movement or accidental drops could compromise a safety-critical procedure.

### 4.3. Grip Closure Control (The Egg)

**Figure 5:**
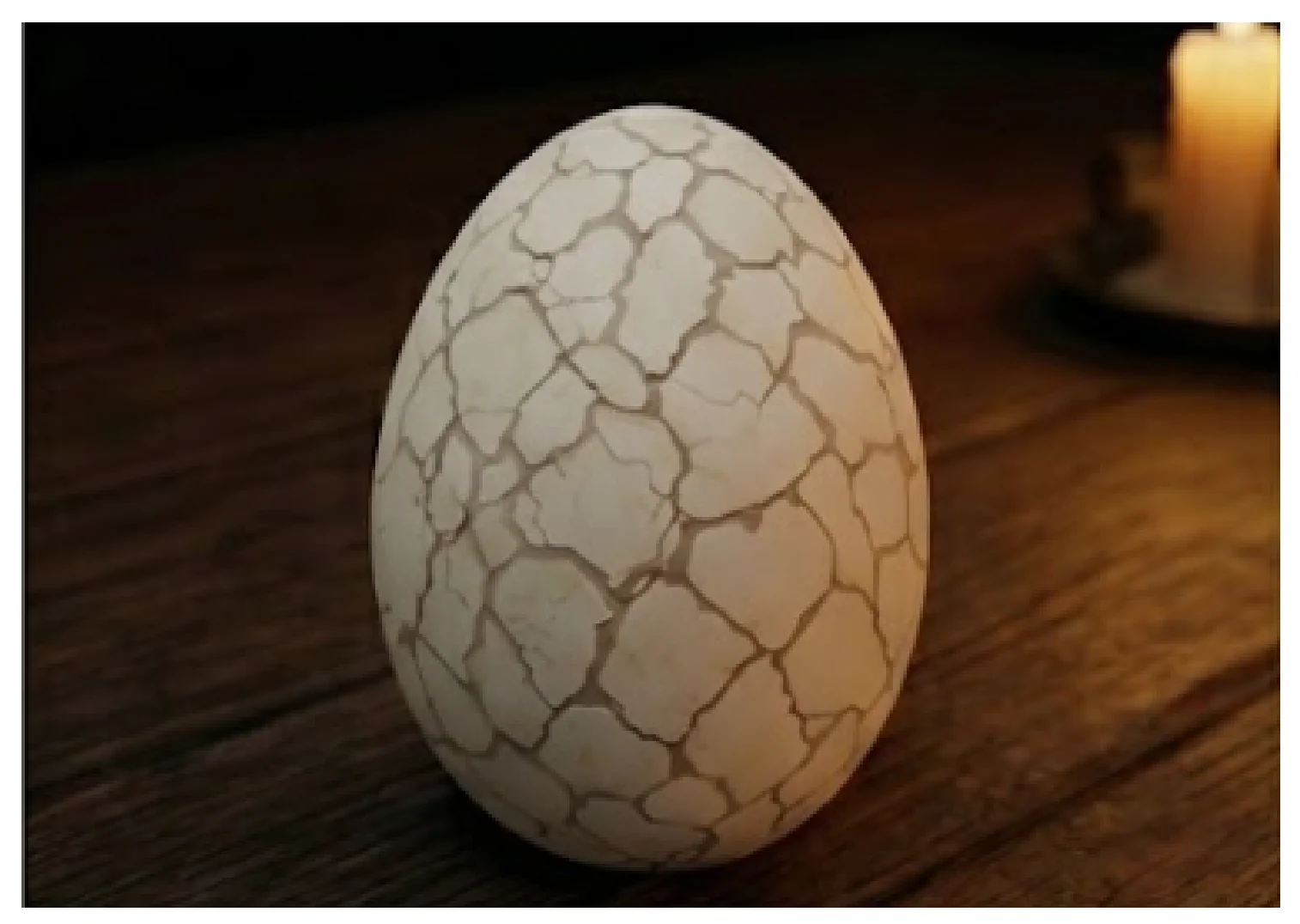
Egg.

To evaluate the user’s ability to control grip closure on a brittle object, we tested the differences in manipulation of a virtual egg (see Figure 5).

This object is modeled so as to maintain structural integrity only as long as the penetration depth of the finger colliders remains below a break threshold. On contact, the glove applies a constant braking command of 0.2 (normalized force-feedback command, 0–1, where 1 corresponds to the maximum braking force), i.e., 20% of the maximum braking force; the egg breaks when the finger colliders penetrate more than 5 mm into its collider (the SDK’s yield distance). Both values were selected following preliminary testing, as they provided the most effective balance between perceived structural fragility and the device’s mechanical constraints. Upon failure, the object *shatters* and the resistance provided by the glove is instantaneously released. This sudden loss of force feedback physically simulates the structural collapse of the shell, requiring the user to control finger closure by relying on haptic cues and input granularity. After an accidental break, the egg respawned at its initial position and the participant could retry. No time limit or maximum number of attempts was imposed: participants decided autonomously when to stop retrying, and the task was recorded as unsuccessful if they stopped without achieving a break inside the target zone. This task is conceptually analogous to graded grip closure required in rehabilitation exercises involving grasping [16,42] or in surgical palpation, where excessive compression risks damaging delicate tissue. It is worth noting that the task measures the user’s control over virtual finger penetration, which acts as a proxy for, but is not a measurement of, the physical grip force exerted by the user.

### 4.4. Compliant Object Grasping (The Soft Snake)

**Figure 6:**
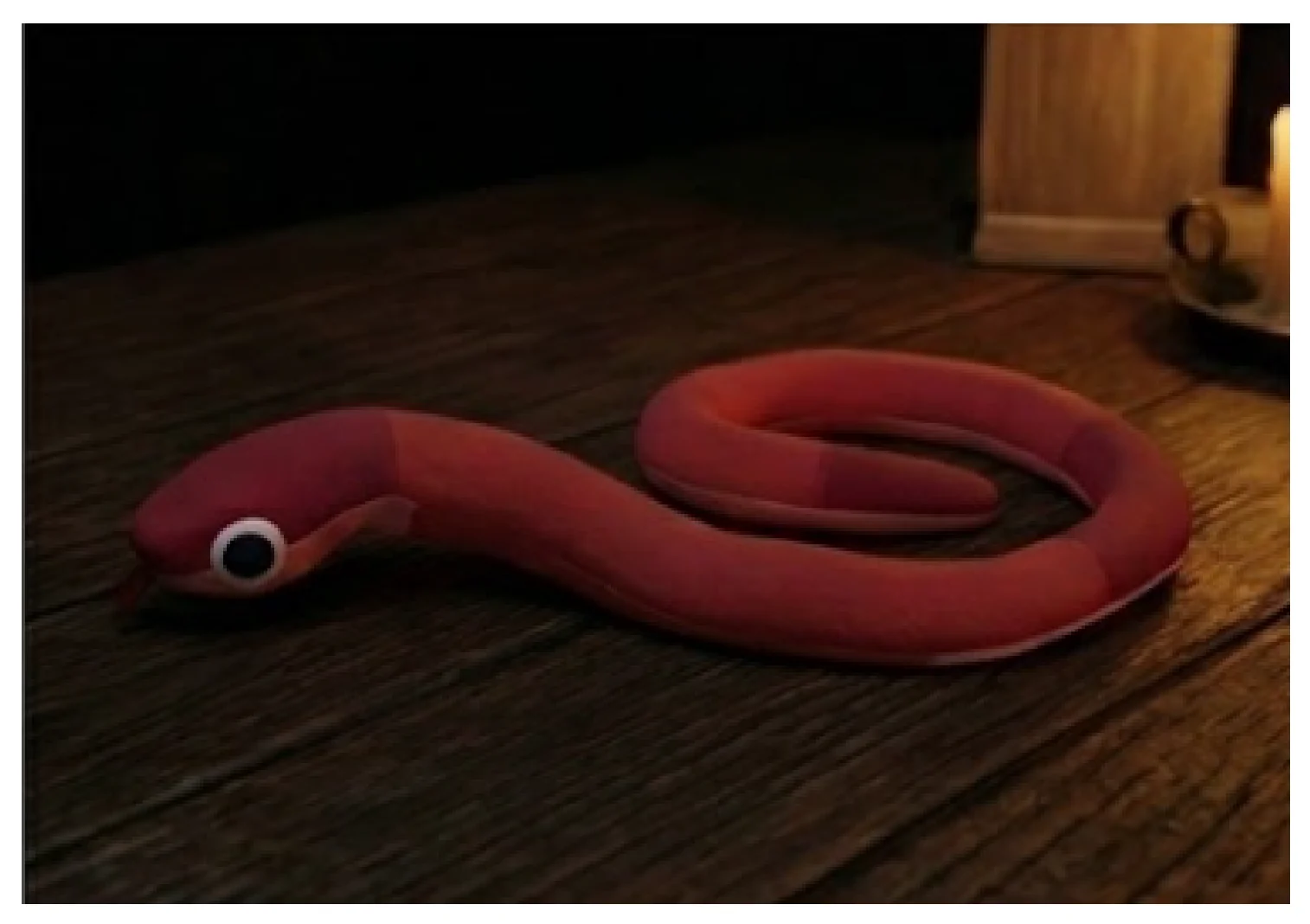
Soft Snake.

The snake (see Figure 6) features a gradual *force displacement profile*, where resistance increases with penetration depth to simulate elasticity. The normalized force-feedback command increases linearly with penetration depth, reaching its maximum at 30 mm.

Hence, the user’s real fingers can continue flexing slightly even after coming into contact with the virtual surface. This scenario examines whether users can maintain a stable grasp on a compliant object without letting it slip and is conceptually related to the manual handling of soft biological tissue or flexible medical instrumentation (e.g., catheters, tubing), where compliance cues guide grip modulation to prevent slippage or deformation.

## 5. User Study

To quantify the operational impact of the hybrid configuration, we conducted a *within-subjects* user study taking the standard system as baseline vs. the proposed hybrid system.

### 5.1. Experimental Design

The study followed a *within-subjects design*, requiring each participant to perform the complete set of tasks using the two distinct experimental setups described in Section 3:

- **Standard System (Baseline):** Interaction utilizing only the commercial haptic glove (see Section 3.1).
- **Hybrid System:** Interaction utilizing the proposed augmented architecture, comprising the data glove and the haptic glove (see Section 3.2)

In order to mitigate learning effects and order bias, the sequence of conditions was counterbalanced. Participants were randomly assigned to two groups: **Group A** (n=9) started with the Standard system, while **Group B** (n=9) started with the Hybrid system. Given the study’s focus on fine manipulation skill, participants were stratified into two groups based on self-reported VR familiarity [43], allowing us to account for the *expertise reversal effect* [44], where the impact of system fidelity is expected to vary based on the user’s pre-existing sensorimotor maps for virtual interaction:

- **High-experience (n=9):** Users who interact with VR regularly or reported extensive prior experience (≥50 total lifetime hours).
- **Low-experience (n=9):** Users with no prior to sporadic exposure to VR (<50 total lifetime hours).

### 5.2. Metrics

Both objective performance and subjective user ratings were collected.

#### 5.2.1. Objective Metrics

- **Missed Grasps:** Number of failed attempts to grab an object (within the target object’s bounding box).
- **Accidental Breaks:** Number of times the Glass Vial or Egg broke unintentionally before task completion (for the Egg, outside the target zone).
- **Successful Breaks:** Specific to the Egg task, this indicates whether the user successfully triggered the fracture mechanism within the target zone.
- **Completion Time:** The total time in seconds to complete the three-task sequence.

#### 5.2.2. Subjective Metrics

- **Workload (Raw NASA-TLX):** We used the Raw NASA-TLX [45] to assess the perceived workload across six dimensions (Mental, Physical, Temporal Demand, Performance, Effort, Frustration).
- **Sense of Agency (SoA):** To measure the feeling of control, we adapted items from the *Agency and Motor Control* subscale of the standardized embodiment questionnaire [46]. Participants rated statements such as “The virtual hand moved exactly as I intended” on a 7-point Likert scale.
- **Preference:** A final comparative questionnaire asked: “With which system did you feel greater precision?” and “Which system provided better haptic feedback?”. Participants could choose between “Standard”, “Hybrid”, or “Equal”.

### 5.3. Participants

We recruited 18 participants (n=18; 13 men, 5 women) aged between 18 and 44 (M=26.28, SD=5.37), with backgrounds in Computer Science, Artificial Intelligence, and Design. All reported normal or corrected-to-normal vision, full hand dexterity, and provided written informed consent per institutional guidelines.

### 5.4. Procedure

**Figure 7:**
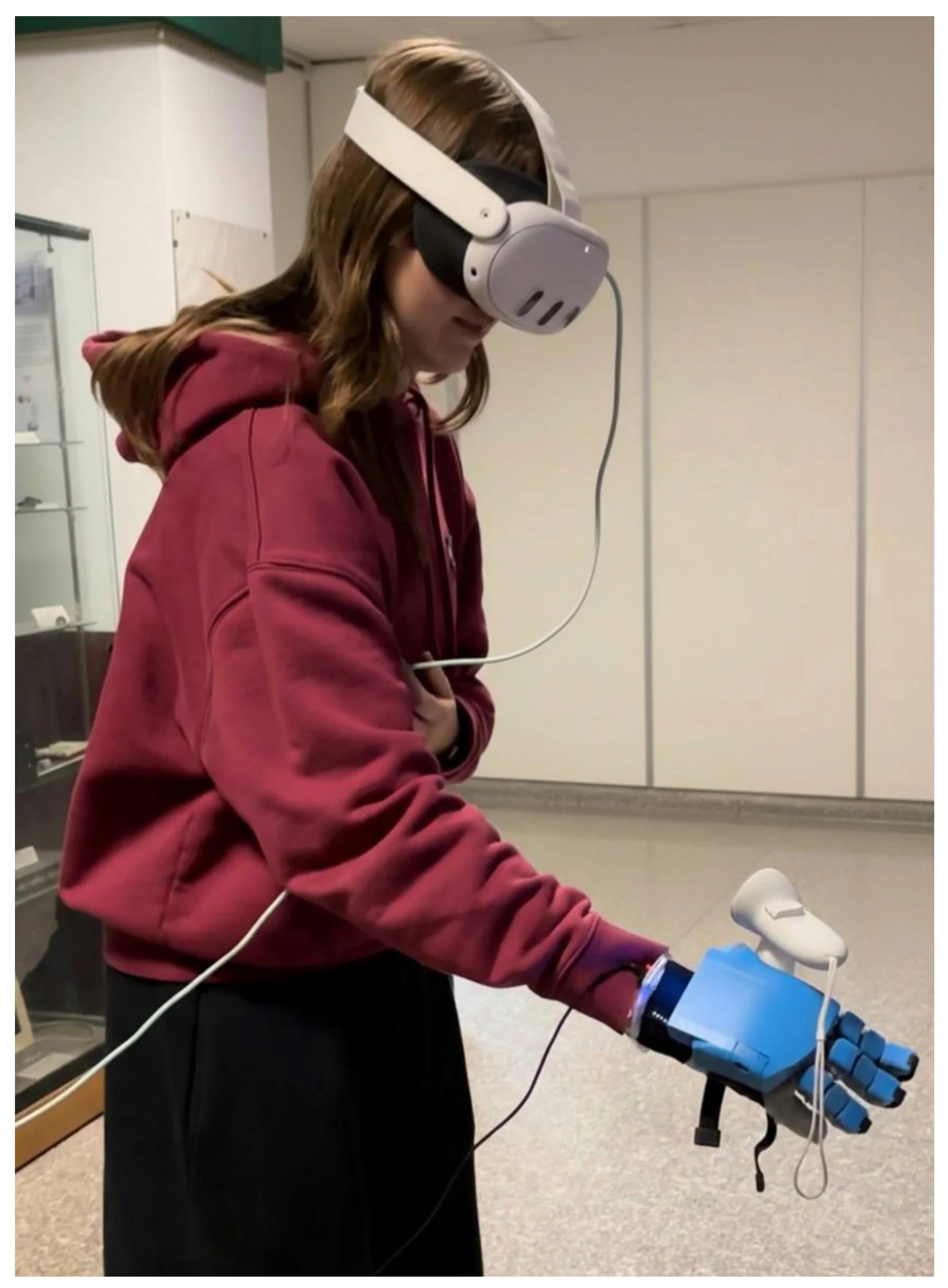
A participant performing the experimental tasks.

The experimental session lasted approximately 20 min per participant. Upon arrival, participants were briefed on the tasks and equipped with the hardware (see Figure 7). Depending on their assigned group, they proceeded with the first system.

- **Calibration (Hybrid only):** For the Hybrid system, a brief calibration (approximately 20 s) mapped the data glove’s carbon nanotube sensors to the user’s hand dimensions. Conversely, the Standard system required no per-user software calibration, since we relied on the SDK’s default calibration profile combined with the proper mechanical adjustment of the glove’s straps, which in preliminary tests provided adequate tracking across all participants’ hand sizes. This procedural asymmetry stems directly from the hardware architectures described in Section 3.2: while the Nova 1’s external cable system mechanically accommodates anatomical differences, the textile-embedded CNT sensors require software normalization to minimize kinematic cross-coupling and alignment errors across different hand sizes [47].
- **Task Execution:** Participants performed the three tasks in a fixed order (Vial → Egg → Snake).
- **Questionnaires:** Immediately after completing the experimental tasks, participants removed the HMD and filled out the *Raw NASA-TLX* and the *SoA* questionnaires.

After a short break, they repeated the entire procedure with the second system. Finally, after completing both sessions, they answered a comparative survey regarding their preference.

## 6. Results

For data analysis and visualization we adopted the Python language (v3.10.15), in particular using the NumPy (v1.26.4), Pandas (v2.2.2), and Matplotlib (v3.9.2) libraries. In this section, we present the quantitative and qualitative findings derived from the user study.

### 6.1. Objective Performance

Table 1 summarizes the interaction metrics. For the Glass Vial task, the Hybrid system showed a reduction in missed grasps for both groups (from 5 to 0.56 in the Low group, and from 4.22 to 0 in the High-experience group), with zero accidental breaks in both configurations. The Fragile Egg task revealed a trade-off: while the Hybrid system increased missed grasps overall, Low-experience users recorded fewer accidental breaks with the Hybrid system (0.89 vs. 1.22). Task success on the Egg differed by experience level: Low-experience users succeeded more with the forgiving Standard system, whereas among High-experience users one participant achieved a successful break with the Hybrid setup and none with the Standard system.

Regarding completion time (Table 2), Low-experience users averaged 86.33 s with the Standard system and 99.33 s with the Hybrid system, while High-experience users recorded 51.11 s and 61.11 s respectively, showing a small increase in task duration across groups when using the Hybrid setup.

### 6.2. Workload (Raw NASA-TLX)

**Figure 8:**
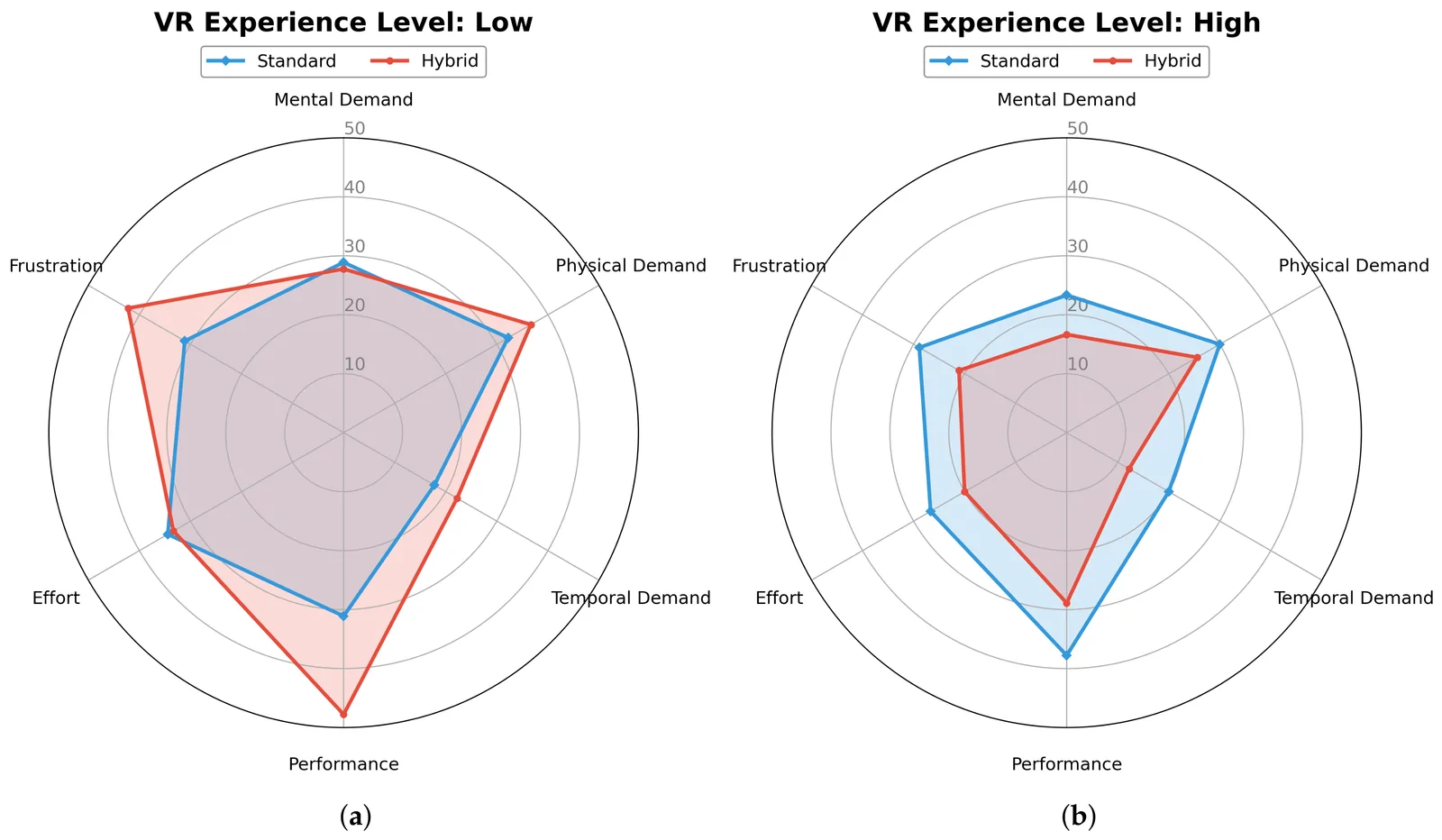
NASA-TLX workload profiles across six dimensions, comparing the Standard and Hybrid systems, stratified by user VR experience level. (**a**) Comparison for users with Low-experience. (**b**) Comparison for users with High-experience. Mean scores reflect the unweighted (Raw-TLX) 0–100 scale; radar axes are scaled to 50 for visual clarity.

Perceived workload (Table 3) followed an inverse trend to user experience. Users in the Low-experience group found the Hybrid system more demanding (27.78 vs. 21.40), driven by increases in perceived workload dimensions (Figure 8a). Conversely, High-experience users reported a lower workload with the Hybrid system (11.93 vs. 19.75), with lower scores on Performance and Mental Demand (Figure 8b). The combined metrics reflect balanced average workloads across configurations (19.86 vs. 20.58).

### 6.3. Sense of Agency (SoA)

The Sense of Agency (Table 4) mirrored the workload’s divergent pattern. An overall improvement with the Hybrid system was observed firstly among High-experience users, who reported an increase in their sense of control (1.36 vs. 0.36). Conversely, the Low-experience group showed little change compared to the Standard baseline (0.78 vs. 0.75).

### 6.4. User Preference

**Figure 9:**
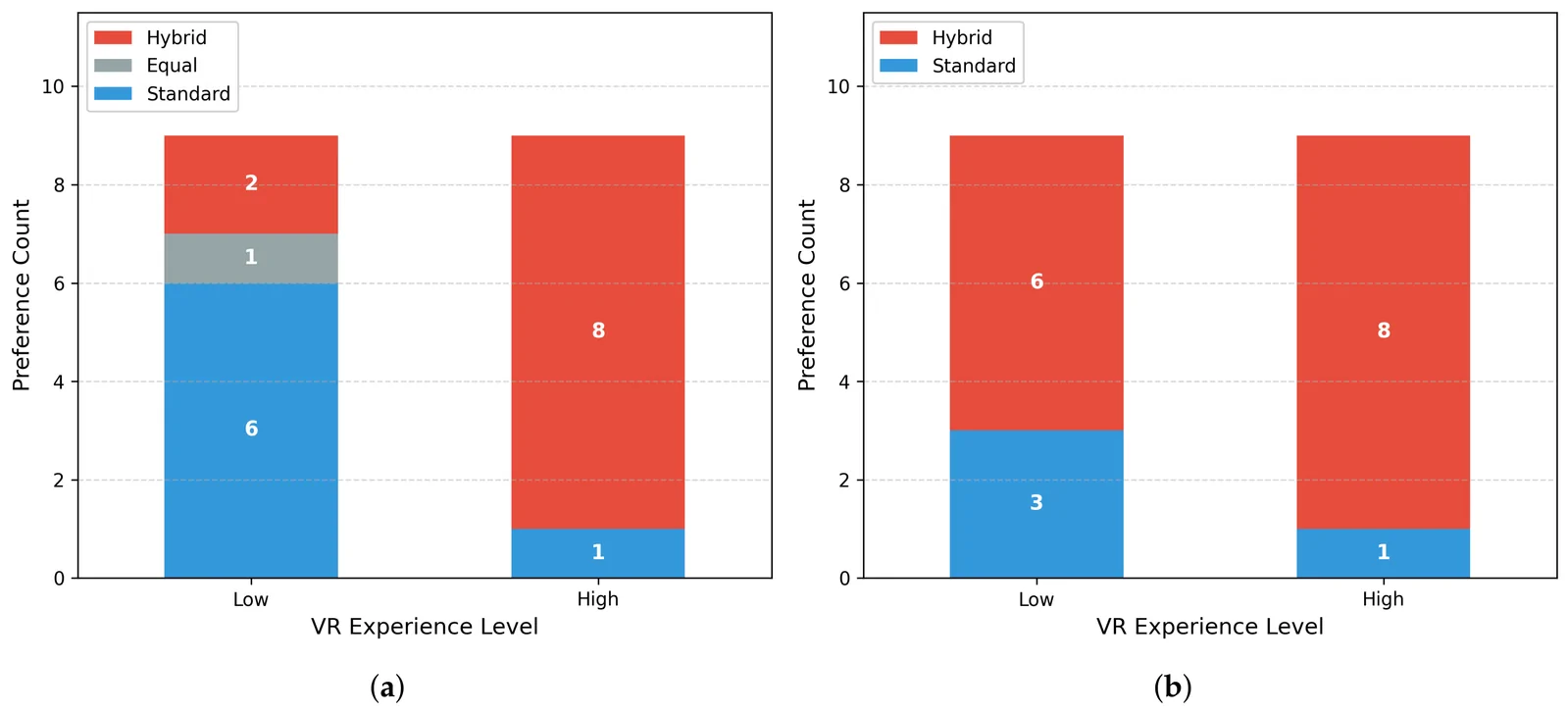
User preference for system precision and haptic feedback, stratified by VR experience level. (**a**) Based on perceived precision. (**b**) Based on perceived quality of haptic feedback.

Preferences revealed a dichotomy based on prior experience. Low-experience users preferred the Standard system for precision, while High-experience users favored the Hybrid system (Figure 9a). Despite both setups using identical force-feedback hardware, the majority (14 out of 18) preferred the haptic feedback of the Hybrid system (Figure 9b).

### 6.5. Statistical Significance Analysis

To complement the descriptive statistics reported in Section 6.1, Section 6.2, Section 6.3 and Section 6.4, paired significance tests were conducted for each objective and subjective metric, comparing the Standard and Hybrid systems within each VR experience group. Given the within-subjects design, comparisons were paired. The Wilcoxon signed-rank test was used uniformly for all count-based and continuous metrics (Table 5 and Table 6), rather than conditioning the test choice on a preliminary normality check: with subgroups of n=9 or fewer, the Shapiro-Wilk test has limited power to detect departures from normality, and a two-stage decision procedure of this kind does not have well-characterized error-rate properties. Given the small number of pairs and the presence of tied differences, a common occurrence with count data, exact *p*-values were computed from the permutation distribution of the signed-rank statistic, which remains valid in the presence of ties. The rank-biserial correlation *r* is reported as effect size, consistent with the non-parametric nature of the test. It was computed as the matched-pairs rank-biserial correlation $r=\left(W^{+}-W^{-}\right)/\left(W^{+}+W^{-}\right)$, where $W^{+}$ and $W^{-}$ are the sums of the ranks of positive and negative differences; zero differences were discarded and tied absolute differences received average ranks. The reported statistic *W* is $\min(W^{+},W^{-})$. The effect size does not depend on the method used to compute *p*. McNemar’s exact test was used for Successful Breaks, a binary outcome, for which no comparable effect size is reported. The association between VR experience level and user preference was tested with Fisher’s exact test, given the small expected cell counts in several comparisons (Table 7); Cramér’s *V* is reported as an effect size for these associations.

Table 5 summarizes the tests for objective performance metrics. None of the paired comparisons reached conventional significance (p<0.05). Notably, six of the eight Wilcoxon comparisons (both Glass Vial Missed Grasps groups, Egg Missed Grasps and Accidental Breaks in the High-experience group, and Soft Snake Missed Grasps in both groups) involved five or fewer non-zero-difference pairs, a sample size at which p<0.05 cannot be reached regardless of the observed effect. This was most evident for Glass Vial Missed Grasps in the High-experience group ($n_{nz}=5$, p=0.063), which reached the lowest *p*-value obtainable at this sample size despite a maximal effect size (r = 1.00, with all non-zero differences favoring the Hybrid system); this result should therefore be read as consistent with, but not statistically confirming, a difference between systems. Glass Vial Accidental Breaks showed zero variance in both conditions for both groups and was therefore not tested.

Table 6 summarizes the tests for completion time and subjective metrics. Unlike several objective comparisons, all these metrics retained at least eight non-zero-difference pairs, placing p<0.05 within reach in principle; nonetheless, none of the paired comparisons for NASA-TLX, Sense of Agency, or Completion Time reached significance in either experience group (all p≥0.15). With *n* = 9 per group, the tests had 80% power only for large effects ($d_{z}\geq 1.07$, sensitivity analysis for a two-sided paired *t*-test at α=0.05, requirements for the Wilcoxon test are slightly higher); non-significant results should therefore not be read as evidence of equivalence.

Table 7 summarizes the association between VR experience level and user preference. The association between experience level and precision preference was statistically significant (Fisher’s exact p=0.020, Cramér’s V=0.67), while the association between experience level and haptic feedback preference was not (p=0.577, Cramér’s V=0.27).

### 6.6. Qualitative Observations

Although not captured by quantitative metrics, direct observation of user behavior provided additional context. Specifically, we noted that users with high VR experience initially attempted to use ’gamified’ strategies (e.g., exploiting collision tolerance to move rapidly) with the Standard system. However, when using the Hybrid system, these users visibly adjusted their posture and handling speed, interacting with the virtual objects more cautiously, akin to handling delicate physical objects. Participants informally reported (no dedicated questionnaire item was used) that the double-gloving configuration did not cause perceived bulkiness or restrict their natural range of motion, and no visible slippage between the two layers was observed by the experimenters during the session.

## 7. Discussion

We examine the results from different angles, also with respect to indications for suitability to assistive applications.

### 7.1. Assisted Accessibility vs. Instrumental Precision

Before interpreting these results, we note that, given the sample size, most of the individual comparisons discussed in Section 6.5 did not reach conventional statistical significance. The following discussion should therefore be read as an interpretation of consistent descriptive and qualitative trends reflecting differences between the two system configurations as a whole, rather than as confirmed performance differences.

The divergent performance patterns observed in Section 6 suggest that the two systems operate under different interaction paradigms. We interpret the standard system as providing ’assisted accessibility’ (where signal noise creates a tolerance for imprecise inputs) whereas the hybrid system offers ’instrumental precision’, enforcing a strict, unforgiving mapping of physical movements to the virtual space. We hypothesize that commercial haptic gloves rely on signal noise to create an invisible layer of assistance. As suggested by the reduction in missed grasps in the Glass Vial task (Table 1), the Hybrid architecture appears to reduce **signal ambiguity**, as reflected in objective metrics. However, the data regarding missed grasps (particularly with rigid objects like the Egg) reveals the double-edged nature of this cleanliness. We hypothesize that, in the Standard setup, the intrinsic imprecision creates a “volumetric” contact rather than a punctual one. This noise allows users to trigger a grasp even with imperfect finger positioning (essentially masking the limitations of rigid-body physics engines which lack the compliance of human skin). The Hybrid system appears to remove this invisible assistance. By enforcing strict adherence to the simulation’s geometry, it may act as a rigorous filter: if the user does not position their fingers with exact precision, the grasp is not triggered. This behavior may explain the polarization of performance observed in our study: by removing the safety net, the system exposes the raw gap between a successful technique and a failed one. While this penalizes the immediate scores of Low-experience users, it suggests that there is room for improvement for the Hybrid setup as a possible *professional instrument*. Unlike the commercial baseline, which prioritizes ease of use through tolerance, the Hybrid system may enforce a stricter correspondence between hand posture and virtual contact, potentially distinguishing between an approximate movement and a correct one: a critical prerequisite not only for advanced training but also for precise assistive environments and functional motor monitoring.

One possible interpretation is that the variations in **completion time** (Table 2) reflect a *behavioral adaptation* rather than a loss of efficiency, a reading supported by our qualitative observations. For Low-experience users, the increase in task duration with the Hybrid system may reflect a shift from hasty and approximate movements (permitted by the tolerance of the Standard system) to a careful alignment of fingers. We observed users pausing to verify their grasp, consistent with a “quality over speed” strategy. This behavioral shift was also evident in High-experience users, who slowed down slightly, possibly due to increased realism. As noted in our qualitative observations (Section 6.6), high-experience users were seen abandoning “gamified” strategies (exploiting software tolerances to move fast) in favor of slower, more cautious handling of the delicate virtual objects. Rather than treating time efficiency as the sole metric of success, these behavioral adaptations suggest that the Hybrid configuration may encourage deliberate movement patterns, which is an important aspect in assistive and rehabilitative scenarios where movement quality and spatial accuracy outweigh raw speed.

The subjective **workload** analysis (Table 3, Figure 8) points to a distinct positioning of the two systems. The Standard system, with its lack of calibration and inherent error tolerance, prioritizes accessibility, serving as a low-stress “plug-and-play” solution for users with limited experience. In contrast, the Hybrid system imposed a clear barrier to entry: the requirement of an initial calibration phase, combined with the unforgiving nature of the grasping mechanics, may have acted as stressors for Low-experience users (driving up *Frustration*). We speculate that users in this group may have attributed these strict requirements to system failures rather than to their own technique. However, for users who already possess the necessary motor skills, the Hybrid configuration may remove the burden of compensating for tracking noise, allowing them to operate at their maximum potential and reducing perceived workload. These findings carry important design considerations for assistive and interactive technologies: adopting precision-oriented tracking may entail a trade-off, imposing a steeper initial learning curve to unlock superior performance and lower cognitive friction for advanced or proficient users.

This divergence extends to the **sense of agency** (Table 4). High-experience users, who may possess a more refined sensitivity to proprioceptive drift, reported a higher sense of control with the Hybrid system, suggesting an improved perception of embodiment compared to the baseline. In contrast, Low-experience users did not perceive this variation, suggesting that the potential benefits of the Hybrid configuration are only appreciable once the user has mastered the basic interaction mechanics.

Finally, user preferences regarding **haptic quality** (Figure 9) highlight a notable psychophysical pattern. While preference for precision was split based on experience, most participants converged in their preference for the Hybrid system’s feedback quality. The majority of participants rated the Hybrid system as superior, despite the force-feedback hardware being identical in both configurations. A possible explanation is that perceived haptic quality depends on the coherence between visual and haptic contact. The brain integrates vision and touch into a single percept: if the Hybrid configuration improved spatial synchronization, this may have created a coherent sensory illusion. In the Standard system, even small misalignments (jitter) may disrupt this coherence, causing the same mechanical force to be perceived as less accurate. An alternative or complementary explanation is that the double-gloving configuration (inner fabric glove and plastic liner) slightly damped the braking force or altered its perceived onset; although participants reported no perceived bulkiness (Section 6.6), this contribution cannot be excluded with the present data.

### 7.2. Extending Assistive and Psychomotor Training Applications

As discussed in Section 7.1, the value of the hybrid architecture may lie not only in delivering force feedback, but potentially in reducing the interaction tolerance that allows imprecise technique to go undetected. The three manipulation profiles used in this study were chosen to approximate distinct classes of clinically relevant handling challenges—rigid delicate objects, brittle materials sensitive to excessive closure, and compliant tissue-like surfaces. This has a direct implication for medical and psychomotor training systems in general: tools such as our own ACSB platform [2], the Yamaha-glove exergame framework [34], and our tactile communication devices [14,15] relied either on precision-oriented tracking alone, or on force feedback layered onto noisy commercial sensing, without the ability to reliably distinguish a correct execution from an approximate one. Our exploratory results suggest that decoupling tracking from force-feedback delivery could help move such systems beyond passively replicating movement toward assessing technique—flagging excessive closure, incorrect grip, or unstable handling in real time, rather than only recording that an action occurred. This shift appears relevant for contexts where the explicit goal is not task completion but the acquisition of genuine motor competence.

### 7.3. Implications for Force-Feedback-Augmented Motor Rehabilitation

This principle finds a particularly concrete application in our prior FES-based rehabilitation framework [16], in which finger flexion recorded from a reference hand via a CNT-instrumented data glove is mapped to FES-evoked mirror movements of a target hand, without any resistance or applied-force dimension. Introducing a haptic glove, as explored in the present study, into this framework could add a controllable and quantifiable force dimension to both sides of the bilateral interaction, complementing rather than replacing the existing kinematic mapping. Two directions appear particularly promising. First, equipping the affected hand with a haptic glove would allow FES-assisted or voluntary grasping exercises to be performed against a controlled, measurable resistance, supporting a progression from unconstrained movement recovery toward graded grip-strength training. This is consistent with prior evidence that combining electrically stimulated grasping with real-time force feedback supports voluntary grip-force control in patients with upper-limb disability [42], and with a recent randomized controlled trial reporting greater improvements in grasping function and grip strength when force feedback was added to hand rehabilitation robot training [48]. Second, equipping the reference (healthy) hand with a haptic glove would allow a clinician to define controlled, repeatable force-feedback conditions that could subsequently inform the FES parameters applied to the target hand, rather than relying solely on unconstrained kinematic mirroring. More broadly, the addition of haptic feedback to stimulation- or exoskeleton-assisted hand rehabilitation has been associated with increased patient engagement and greater activation of motor and somatosensory cortical areas compared to movement-only training [49,50], supporting the rationale for introducing a force-feedback layer into FES-based systems.

A relevant consideration when extending this idea to clinical rehabilitation is that, in our own study, the hybrid system’s stricter, low-tolerance interaction model was better received by high-experience users, while less experienced participants performed and felt better with the more forgiving standard configuration. This distinction may map naturally onto different phases of rehabilitation: a more tolerant, standard-like configuration could better suit early-stage patients still recovering basic motor control, while a precision-oriented, hybrid configuration could support later-stage, force-quantified monitoring of recovery, once finer motor control has been regained. In [16], the CNT glove and the stimulation electrodes are placed on different hands; any configuration co-locating them would require electrical isolation between the two.

### 7.4. Experiment Limitations

The extension outlined above is necessarily speculative, and its feasibility depends on constraints already present in the current study design. We therefore detail these limitations below, several of which carry specific implications for any future clinical deployment.

*Internal limitations* firstly derive from the fact that the per-user calibration phase is required only for the Hybrid system. This setup discrepancy may have acted as a confounding variable, inflating the perceived workload for the Hybrid setup. Additionally, while the fixed sequential task order (Vial → Egg → Snake) was purposefully designed to follow an increasing curve of object deformability and interaction complexity, it introduces potential learning effects across tasks that were not isolated. Moreover, since participants could retry the Egg task until they chose to stop, Accidental Breaks and Completion Time partly reflect individual persistence rather than performance alone, which limits their comparability across participants and conditions. Finally, tracking accuracy (joint-angle error, hysteresis, drift) and end-to-end latency were not measured directly: neither manufacturer reports joint-angle accuracy for these devices, so the advantages of the Hybrid system are inferred from user-level outcomes. These may also reflect other differences between configurations, such as double-gloving, the two-point linear calibration, and the data transmission pipeline.

*External limitations* stem from our sample, consisting of participants with technical backgrounds (Computer Science, Artificial Intelligence and Design), including students. This implies a higher baseline familiarity with technical interfaces, limiting the generalizability of the results to the broader population, including clinical end users. The sample was also gender-imbalanced (13 men, 5 women), and hand size, gaming experience and experience with specialized manual tasks were not recorded. Furthermore, the small sample size and balanced sub-group distributions (Low: 9, High: 9) limit the statistical power of our comparative analyses. Lastly, while we observed differences in user preferences based on experience, the underlying subjective reasons (e.g., familiarity vs. perceived utility) remain speculative and require further empirical validation.

## 8. Conclusions and Future Work

This study suggests a possible trade-off between accessibility and fidelity. The standard system, despite being prone to tracking errors, appeared more permissive with complex geometries, such as the egg, and its “plug-and-play” nature was associated with lower frustration for novice users. However, the Hybrid system was associated with fewer missed grasps in the Glass Vial task and appeared to impose a strict adherence to interaction physics. This rigorous mapping may be a desirable attribute for training simulations. This lack of software tolerance makes manipulating difficult objects more challenging, as it requires precise motor control from the user. This suggests that the hybrid architecture may be a promising choice for assistive technologies and applications, such as rehabilitation, where a simulation must accurately penalize errors rather than facilitate interaction through software tolerance. It is worth noting that, while the descriptive patterns observed throughout this study consistently favor this interpretation, none of the differences in objective or subjective performance (Section 6.5) reached conventional statistical significance given the sample size, with the exception of the association between experience level and precision preference (Table 7); this conclusion should therefore be read as a hypothesis supported by consistent descriptive and qualitative evidence rather than confirmed performance differences, warranting confirmation in future work with larger cohorts.

A steeper learning curve may be acceptable in professional training, which aims to develop genuine motor skills rather than immediate ease of use. Furthermore, our results raise the possibility that the finger-tracking configuration contributes to perceived haptic quality: the majority of participants (14 out of 18) preferred the Hybrid system’s haptic feedback even though the force-feedback hardware was identical in both configurations, suggesting that the hybrid configuration may have improved visuo-haptic coherence, although a contribution of double-gloving cannot be excluded.

Future research will address the limitations identified in Section 7.4. We plan to expand the participant cohort to include a balanced number of users across all experience levels, ensuring broader generalizability for novice populations. To mitigate the setup overhead of the Hybrid system, we will investigate automated, transparent calibration procedures to improve the user experience. Additionally, we plan to integrate the dedicated wireless extension module for the precision-oriented tracking layer, eliminating the current tethered connection. This would enhance freedom of movement and facilitate easier hardware integration for complex simulations. Future studies will also incorporate structured follow-up interviews to gather qualitative insights that explicitly explain the underlying reasons behind users’ system and haptic preferences. Moreover, both configurations will be validated against an optical motion-capture reference, measuring joint-angle error, hysteresis, jitter and end-to-end latency of both acquisition pipelines.

## Figures and Tables

**Table 1:** Performance metrics for the three manipulation tasks, comparing the Standard and Hybrid systems across user VR experience levels. All values are reported as Mean (Standard Deviation). For Successful Breaks (binary), the mean corresponds to the proportion of participants who succeeded.

		Low-Exp. (n=9)		High-Exp. (n=9)	
Virtual Object	Metric	Standard	Hybrid	Standard	Hybrid
**Glass Vial**	Missed Grasps	5.00 (12.03)	**0.56 (1.13)**	4.22 (6.96)	**0 (0)**
	Accidental Breaks	0 (0)	0 (0)	0 (0)	0 (0)
**Egg**	Missed Grasps	**1.33 (1.50)**	6.33 (11.51)	**1.00 (1.73)**	2.78 (7.97)
	Accidental Breaks	1.22 (2.64)	**0.89 (1.45)**	**0.33 (0.71)**	1.11 (2.67)
	Successful Breaks	**0.33 (0.50)**	0 (0)	0 (0)	**0.11 (0.33)**
**Soft Snake**	Missed Grasps	0.11 (0.33)	**0 (0)**	0.56 (1.01)	**0.11 (0.33)**

**Table 2:** Comparison of the Total Mean Completion Time (in seconds) to complete the three task sequence between the two systems across user VR experience levels. Data is reported as Mean (Standard Deviation).

VR Experience Level	Standard	Hybrid
Low	86.33 (31.29)	99.33 (32.31)
High	51.11 (33.47)	61.11 (19.60)
**Overall**	**68.72 (36.28)**	**80.22 (32.54)**

**Table 3:** Comparison of Raw NASA-TLX scores between the two systems across user VR experience levels. Data is reported as Mean (Standard Deviation).

VR Experience Level	Standard	Hybrid
Low	21.40 (15.40)	27.78 (15.32)
High	19.75 (19.13)	11.93 (6.82)
**Overall**	**20.58 (17.38)**	**19.86 (14.26)**

**Table 4:** Sense of Agency scores for the Standard and Hybrid systems, stratified by user VR experience level. Scores are reported on a scale from −3 (no agency) to +3 (full agency) as Mean (Standard Deviation).

VR Experience Level	Standard	Hybrid
Low	0.75 (1.35)	0.78 (1.11)
High	0.36 (1.18)	1.36 (1.17)
**Overall**	**0.56 (1.28)**	**1.07 (1.17)**

**Table 5:** Paired significance tests comparing Standard and Hybrid systems, stratified by VR experience level (Table 1). Given the small samples and the presence of ties among paired differences (common with count data), exact *p*-values were computed from the permutation distribution of the Wilcoxon signed-rank statistic. $n_{nz}$ denotes the number of non-zero-difference pairs used by the test. The rank-biserial correlation *r* is reported as effect size; positive *r* indicates the Standard system had higher counts (i.e., the Hybrid system performed better) for these error-based metrics. With $n_{nz}\leq 5$, p<0.05 cannot be reached regardless of effect magnitude; such cases are marked with †. Glass Vial Accidental Breaks showed zero variance in both conditions and was not tested.

		Low Exp. (n=9)			High Exp. (n=9)		
Virtual Object	Metric	$n_{\mathrm{nz}}$	Test, *p*	*r*	$n_{\mathrm{nz}}$	Test, *p*	*r*
**Glass Vial**	Missed Grasps	5 ^†^	W=2.00, p=0.250	0.73	5 ^†^	W=0.00, p=0.063	1.00
	Accidental Breaks	–			–		
**Egg**	Missed Grasps	6	W=6.00, p=0.406	−0.43	4 ^†^	W=5.00, p=1.000	0.00
	Accidental Breaks	6	W=10.00, p=1.000	−0.05	4 ^†^	W=3.50, p=0.750	−0.30
	Successful Breaks	McNemar, p=0.250			McNemar, p=1.000		
**Soft Snake**	Missed Grasps	1 ^†^	W=0.00, p=1.000	1.00	4 ^†^	W=2.00, p=0.500	0.60

^†^ With $n_{nz}\leq 5$ non-zero-difference pairs, p<0.05 cannot be mathematically reached.

**Table 6:** Paired significance tests for completion time and subjective metrics, comparing Standard and Hybrid systems across user VR experience levels (Table 2, Table 3 and Table 4). The Wilcoxon signed-rank test (exact permutation *p*-values) was used uniformly, consistent with the objective metrics (Table 5). $n_{nz}$ is the number of non-zero-difference pairs. The rank-biserial correlation *r* is reported as effect size; positive *r* indicates higher values for the Standard system.

	Low-Exp. (n=9)			High-Exp. (n=9)		
Metric	$n_{\mathrm{nz}}$	Test, *p*	*r*	$n_{\mathrm{nz}}$	Test, *p*	*r*
**Completion Time**	8	W=13.00, p=0.546	−0.28	9	W=16.00, p=0.476	−0.29
**NASA-TLX**	9	W=10.00, p=0.152	−0.56	8	W=9.00, p=0.250	0.50
**Sense of Agency**	8	W=16.50, p=0.852	0.08	9	W=11.00, p=0.199	−0.51

**Table 7:** Association between VR experience level and system preference (Figure 9). Given small expected cell counts in several comparisons, Fisher’s exact test (Freeman-Halton extension for the 2×3 table) is reported as the primary test; Cramér’s *V* is reported as effect size. Note: In Haptic Feedback Preference, the “Equal” category was excluded from this comparison as no participant selected it in either experience group.

Comparison	Test	*p*	Cramér’s *V*
Precision preference (Standard/Equal/Hybrid) × Experience	Fisher exact (2×3)	0.020	0.67
Haptic feedback preference (Standard/Hybrid) × Experience	Fisher exact (2×2)	0.577	0.27

## Data Availability

The data presented in this study are available on request from the corresponding author. The data are not publicly available due to institutional data-sharing policies.

## Abbreviations

The following abbreviations are used in this manuscript:

ACSB	Anesthesia Crisis Scenario Builder
CNT	Carbon Nanotube
DoF	Degrees of Freedom
FES	Functional Electrical Stimulation
GF	Gauge Factor
HMD	Head-Mounted Display
IMU	Inertial Measurement Unit
IP	Interphalangeal
PIP	Proximal Interphalangeal
MCP	Metacarpophalangeal
NASA-TLX	NASA Task Load Index
PC	Personal Computer
SDK	Software Development Kit
SoA	Sense of Agency
UDP	User Datagram Protocol
USB	Universal Serial Bus
UX	User Experience
VR	Virtual Reality
